# Prevalence and related factors of inappropriate gestational weight gain among pregnant women with overweight/ obesity in Thailand

**DOI:** 10.1186/s12884-023-05635-0

**Published:** 2023-05-05

**Authors:** Thanyawalai Chairat, Ameporn Ratinthorn, Piyanun Limruangrong, Dittakarn Boriboonhirunsarn

**Affiliations:** 1 Faculty of Nursing, Mahidol University, Bangkok, 10700 Thailand; 2 Department of Obstetrics and Gynecology, Faculty of Medicine Siriraj Hospital, Mahidol University, Bangkok, 10400 Thailand

**Keywords:** GWG, Overweight/obesity, Antenatal service system, Pregnancy, Thailand

## Abstract

**Background:**

An inappropriate gestational weight gain (GWG) among pregnant women with overweight/obesity is a crucial health problem. Its prevalence remains high worldwide, particularly in urban areas. The prevalence and predicting factors in Thailand are lack of evidence. This study aimed to investigate prevalence rates, antenatal care (ANC) service arrangement, predictive factors, and impacts of inappropriate GWG among pregnant women with overweight/obesity in Bangkok and its surrounding metropolitan area.

**Methods:**

This cross-sectional, retrospective study used four sets of questionnaires investigating 685 pregnant women with overweight/obesity and 51 nurse-midwives (NMs) from July to December 2019 in ten tertiary hospitals. Multinomial logistic regression identified predictive factors with a 95% confidence interval (CI).

**Result:**

The prevalence rates of excessive and inadequate GWG were 62.34% and 12.99%. Weight management for pregnant women with overweight/obesity are unavailable in tertiary cares. Over three-fourths of NMs have never received weight management training for this particular group. ANC service factors, i.e., GWG counseling by ANC providers, quality of general ANC service at an excellent and good level, NMs' positive attitudes toward GWG control, significantly decreased the adjusted odds ratio (AOR) of inadequate GWG by 0.03, 0.01, 0.02, 0.20, times, respectively. While maternal factors, sufficient income, and easy access to low-fat foods reduce AOR of inadequate GWG by 0.49, and 0.31 times. In contrast, adequate maternal GWG knowledge statistically increased the AOR of inadequate GWG 1.81 times. Meanwhile, easy access to low-fat foods and internal weight locus of control (WLOC) decreased the AOR of excessive GWG by 0.29 and 0.57 times. Finally, excessive GWG significantly increased the risk of primary C/S, fetal LGA, and macrosomia 1.65, 1.60, and 5.84 times, respectively, while inadequate GWG was not associated with adverse outcomes.

**Conclusion:**

Prevalence rates of inappropriate GWG, especially excessive GWG remained high and affected adverse outcomes. The quality of ANC service provision and appropriate GWG counseling from ANC providers are significant health service factors. Thus, NMs should receive gestational weight counseling and management training to improve women's knowledge and practice for gestational weight (GW) control.

## Background

Inappropriate GWG is a crucial health problem for pregnant women with overweight/obesity because it causes adverse maternal and neonatal outcomes [1, 2]. The global prevalence of overweight and obesity (pre-pregnancy BMI ≥ 25 kg/m^2^) in women of reproductive age has increased continuously in Thailand, particularly in Bangkok and its surrounding area [3]. Over 50% of pregnant women with overweight/obesity are more likely to have inappropriate GWG, especially excessive GWG [2, 4] that is associated with adverse outcomes such as pregnancy-induced hypertension (PIH), cesarean delivery, large-for-gestational-age (LGA) fetus and macrosomia [1, 2]. Meanwhile, inadequate GWG is associated with small-for-gestational-age (SGA) neonates [5, 6], low birth weight, and preterm birth [1]. Thus, the Ministry of Public Health, Thailand (MOPH) launched surveillance of appropriate GWG as a process indicator of the ANC service delivery system [7].

According to the determinants of GWG by IOM (2009), inappropriate GWG is a preventable pregnancy outcome that may be influenced by multifaceted factors at multiple levels [8]. The evidence showed that nulliparous, obese pre-pregnancy BMI [4] and ability to access healthy food [8, 9] were significantly associated with inadequate GWG. While several research studies found that maternal characteristics, including medical conditions [4], advanced or younger maternal age, single status, nulliparous [4, 10], received correctly advice about GWG [10], low income [11], overweight pre-pregnancy BMI [4, 8, 12], and inadequate healthy food consumption [12, 13] were significantly related to excessive GWG. Whereas, some maternal factors in those researches evidence were not significantly related to GWG [9, 14, 15]. In addition, the maternal knowledge, WLOC, perceived barriers, social support, and intentions defined as health cognitions [16, 17] were associated with GW control [18–20]. However, the studies found that GWG could result from GWG counseling women received during antenatal care [21, 22].

The ANC service system factors may significantly contribute to inappropriate GWG [8]. The ANC service arrangement, including characteristics and resources in healthcare settings [8, 23], and lack of concerned about GW control from healthcare providers [24] were associated with GWG. Meanwhile, the lack of knowledge, confidence, and negative attitude toward GW control of healthcare providers were associated with providing GWG counseling [25]. The WHO recommended that all pregnant women receive counseling about healthy eating and keeping physical activity (PA) to prevent excessive weight gain during antenatal care visits [26]. The provision of ANC services, such as using standard guidelines and providing effective lifestyle intervention focusing on diet and PA, significantly decreased the percentage of pregnant women with excessive GWG [27–30]. However, some studies reported that brief health behavior interventions did not affect GWG and pregnancy outcomes [31] as well as receiving the fulfilled standard guideline for pregnant women had not affected the pregnancy outcomes [32]. The literature revealed that to achieve appropriate GWG, more intensive personal counseling, follow-up sessions, and individualized adaptive plans are needed. Moreover, the qualitative studies found that women received limited advice about GWG, and conflicting information about IOM recommendations during their antenatal visits [21, 22].

Currently, ANC service arrangement, inappropriate GWG in Thailand, and contributions of maternal and health service system factors to inadequate and excessive GWG among pregnant women with overweight/obesity are less studied. Therefore, this study aimed to investigate prevalence rates, ANC service system arrangement, predictive factors, and impacts of inappropriate GWG among pregnant women with overweight/obesity across 10 tertiary hospitals in Bangkok and Metropolitans area, Thailand. The study results might help develop strategies to promote optimal GWG and effective ANC service delivery for pregnant women.

## Methods

### Study setting, sample size, and design

**Fig. 1:**
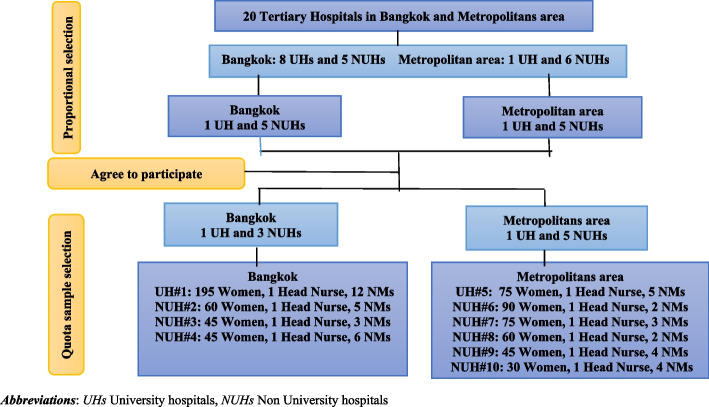
Flowchart of hospital setting and subjects selection

The retrospective and cross-sectional study design were conducted from July to December 2019 in Bangkok and four provinces of the Metropolitan area. The research settings were 10 ANC units (4 ANC units in Bangkok and 6 ANC units in the Metropolitans area). The participants included postpartum women, Head Nurses and NMs of ANC units. The inclusion criteria for postpartum women were age older than 18 years, pre-pregnancy BMI ≥ 25 kg/m^2^, attended 1^st^ ANC before 28 weeks of gestation, singleton pregnancy, delivery at ≥ 37 weeks’ gestation, able to read and understand Thai language. In addition, all Head Nurses of 10 ANC units and 46 full-time NMs at ANC units were eligible. The sample size of postpartum women calculation was based on odds ratio from a previous study [4], using the G*Power program version 3.1 according to correlation and logistic regression models [10, 33], with a *p*-value of 0.05 (2 sided) and power of analysis of 0.80. Moreover, 10% was added to the sample size to account for incomplete responses and data errors. Thus, the total sample size for this study was 720 postpartum women. The quota sampling technique was employed. Figure 1 presents the number of postpartum women in each hospital setting according to the live birth rate among ten tertiary hospitals in 2018 [34]. The study was approved by the Human Research for Ethics Committees of the Faculty of Medicine, Siriraj Hospital, Mahidol University, and all hospital settings.

### Variables and instruments

We used four sets of questionnaires to collect the data from the participants, antenatal records, and labor record forms. The maternal record form and ANC service provision questionnaire were developed by the researchers based on the literature review [26–28, 35–37]. The maternal record form was used to collect maternal characteristics (i.e., sociodemographic data, obstetric data, pre-pregnancy BMI, gestational weight and birth outcomes) retrieved from an antenatal and labor records. The questionnaires on health behaviors [12], health cognitions [17], and healthcare provider's questionnaire [25] were modified with permission for this study. Back translation technique [38] was used to ensure content, semantic, and technical equivalence in health cognitions and healthcare providers' questionnaires. Cronbach’s alpha of health behaviors and health cognitions questionnaires were 0.77–0.72 [12] and 0.58—0.79 [17], respectively. Details of the variables and measurements are presented in Table 1.

**Table 1:** Definition and measures of research instruments

Instrument	Variable	Definition/ Data	No. items	Scale	Score			S-CVI, Cronbach α
**1.The maternal record form**	Sociodemographic^a^	maternal age, marital status, and household income						NA
	access to healthy food^a^	ability of pregnant women to access low-fat food (i.e., lean meat, white fish, egg white, dried beans, fresh/frozen vegetables, baked or boiled potatoes) options and distance from their home to the nutritional environment [8, 9, 14, 23]	2	4-point scale; once a month to more than once a week; less than 2 km to more than 6 km	- Access was the sum of two ability measures. Range 2 to 8, higher scores (6–8) categorized as, "easy access to low-fat food."			
	Obstetric data^b^	parity, medical condition, pre-pregnancy BMI, and received GWG advice						
	Gestational Weight Gain^b^	Total Gestational Weight Gain (TGWG) according to the IOM recommendations for the overweight/obese group [8, 10] that calculated from the mother's last weight in kilograms at the time of admission for delivery minuses pre-pregnancy weight		3 categories of GWG; inadequate to excessive		**Total GWG (kilogram) of pregnant women**		
					**Type of GWG**			
					Adequate	7–11.5	5–9	
					Inadequate	< 7	< 5	
					Excessive	> 11.5	> 9	
	-Pregnancy Induce Hypertension (PIH)^b^	a systolic blood pressure reading ≥ 140 mmHg and/or a diastolic blood pressure ≥ 90 mmHg (average of at least 2 measurements taken at least 15 min apart) [2]						
	-LGA^b^	neonatal birthweight > 90^th^ percentile for gestational age [1]						
	-SGA^b^	neonatal birthweight < 10^th^ percentile for gestational age [1]						
	-macrosomia^b^	neonatal birthweight > 4000 g [1]						
**2.The questionnaires on health cognitions and health behaviors**	-Knowledge^a^	the information or understanding of information about healthy eating, physical activity (PA), and weight gain [17]	11	True/False; one response recorded to correct/incorrect	Number of items with correct responses. Range 0–11, higher scores (9–11) categorized as "adequate GWG knowledge"			S-CVI 0.9 -KR21 0.63
	-Weight locus of control^a^	the belief that body weight is under self-control (internal WLOC) or caused by the environment surrounding respondents (external WLOC) [17]	4	5-point scale; strongly disagree to strongly agree	Mean of all items in measure with external items reverse scored. Range 1 to 5, higher scores (18–20) categorized as "internal WLOC"			Cronbach α 0.71
	-Social support^a^	Perceived support from husband or friends regarding healthy eating, physical activity, and gestational weight control [17]	13	5-point scale	A sum of scores from each individual construct. Range 13 to 65, higher scores (50–60) categorized as "high social support"			Cronbach α 0.63
	-Perceived barriers^a^	The respondents' perception on limitations, constraints, or barriers regarding healthy eating and PA [17]	12	5-point scale	A sum of two barrier measures. Range 12 to 60, higher scores (48–60), categorized as "high barriers."			Cronbach α 0.71
	-Intention^a^	An individual's explicit decisions to behave in a certain way concerning healthy eating, PA, and weight gain [17]	7	7-point scale	A sum of scores from each individual construct. Range 7 to 49, higher scores (39–49), categorized as "high intention."			Cronbach α 0.68
	Healthy food consumption^a^	The respondents' health practices that relate to dietary consumption patterns, food preparation, and type of food consumed [12]	28	4-scale; never to always	Range 28 to 112, higher scores (78–112), categorized as "appropriate healthy food consumption."			S-CVI 0.9 Cronbach α 0.85
	Physical Activity^a^	The respondents' health practices that relate to work/job-related activities, housework, hobbies and travel, exercise activity, and relaxation activity [12]	16	4- point scale	Range 16 to 64, higher scores (45–64), categorized as "appropriate PA."			Cronbach α 0.87
**3.The ANC service provision questionnaire**	-Quality of ANC service provision (general/specific)	The level of care provided to pregnant women [39, 40], derived from the calculation of the percentage score regarding ANC services provision in each ANC unit compared with the standard from literature review [26–28, 35–37] The general service provision in ANC unit regarding screening for anemia, thalassemia, parasite, STD and other infectious diseases, preeclampsia screening at every ANC visit, GDM screening at 1^st^ visit and 24–28 weeks of gestational age, provision of supplements, group nutrition education, evaluation of nutrition and weight gain status, recording nutrition graph or weight gain chart, and making ANC appointments	24	2 - point scale; does exist and does not exist	The percentage score of ANC services provision in each ANC unit was calculated, Range 0–100, the quality level of ANC service provision was categorized [39, 40] as follows: very good (meet completely, 100%), good (meet mostly, 80–99%), fair (meet fairly, 50–79%), poor (must be improved, 2–49%)			S-CVI 1 Cronbach α 0.85
	general		10					
	specific	The specific service provision in ANC unit regarding weight and height measurement at 1st ANC visit, weight measurement at every ANC visit, medical prevention for maternal and neonatal complications during pregnancy, individual nutrition education, preeclampsia screening in women with Class III obesity, GDM screening at 1^st^ ANC visit, ultrasound examination, food consumption assessment, referral to a multidisciplinary team for delivery planning, anesthesia consultation, and providing specific intervention for GW control	14					
**4. The healthcare provider's questionnaire**	GWG knowledge^d^	the accuracy and suitability of recommended GWG, food consumption, and physical activity information that healthcare providers provided to pregnant women [25]	11	5-point scale; strongly disagree to strongly agree	Ranged 11 to 55, high mean scores of knowledge (44–55), categorized as " indicated adequate GWG knowledge"			S-CVI 1 Cronbach α 0.81
	Attitude toward GW control^d^	The importance of GWG management in pregnant women with overweight/obesity in terms of discussion, assessment, and assistance [25]	3	5-point scale	Ranged 3 to 15, high mean scores of Attitude toward GW control (14–15), categorized as "positive attitude"			Cronbach α 0.78
	GWG counseling practice^d^	the process of professional relationships between healthcare providers and pregnant women with overweight/obesity for providing specific advice and information regarding GWG, nutrition, dietary consumption, and physical activity [25]	18	5-point scale; almost never to almost	Ranged 18 to 90, high mean scores of GWG counseling practice (63–90), categorized as "routine GWG counseling practice"			Cronbach α 0.79

^a^ Data were obtained from postpartum women who had overweight/obesity pre-pregnancy BMI

^b^ Data were obtained from an antenatal care booklet, and summary of labor record forms

^c^ Data were obtained from 10 Head Nurses

^d^ Data were obtained from 10 Head Nurses and 41 NMs

### Statistical analyses

Descriptive statistics, including frequency, percentage, mean, and standard deviation (SD) were used to summarize the characteristics of postpartum women, NMs, and the ANC service system arrangement. Chi-square test was used to test the differences in the proportions of variables between GWG categories and birth outcomes at a significant level of 0.05. The assumption for statistics use of multilevel logistic regression analysis was tested by using intraclass correlation coefficient (ICC) to see the variance of health service level that contributed to inadequate GWG and excessive GWG that were 0.0265 and 0.0073, respectively. This indicates that there were no variations in hospital levels. The univariate logistic regression analysis was performed and a more conservative level of statistical significance (*p*-value < 0.20) was used in selecting variables in the predictive model [41, 42]. Multivariable multinomial logistic regression models were used for testing the predictive model of inappropriate GWG, using an odds ratio with a 95% confidence interval. Meanwhile, binary logistic regression models were used to verify the effect of inappropriate GWG to maternal and neonatal birth outcomes at a significant level of 0.05. Statistical analysis used the IBM© Statistic Package for the Social Sciences (SPSS), version 20 [43].

## Results

### Prevalence of inappropriate GWG among pregnant women with overweight/obesity

**Fig. 2:**
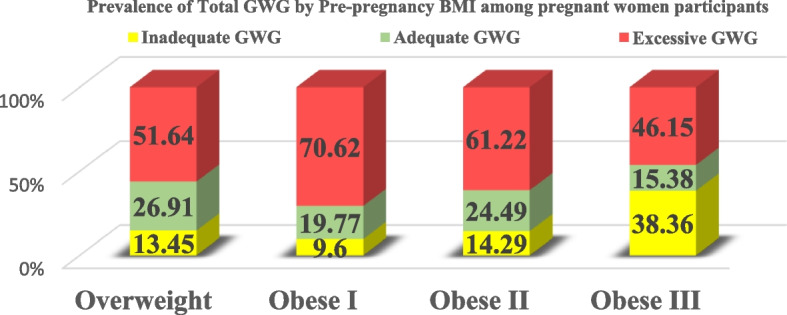
Prevalence of total GWG stratified by pre-pregnancy BMI

Of the 685 postpartum women who participated in the current study, 446(65.12%), 177 (25.84%), 49(7.15%), and 13 (1.89%) were pregnant women with overweight, obese I, obese II, and obese III, respectively. The prevalence of adequate GWG was 26.91%, 19.77%, 24.49, and 15.38%, respectively. Notably, the percentage of excessive GWG decreased, while the share of inadequate GWG increased among obese I, II, and III, respectively (Fig. 2).

### Maternal characteristics and information

Of postpartum women, 685 who completed the questionnaires (from a response rate of 689 mothers, 95.69%) were included in the data analysis. Participants' mean age was 29.76 years (SD = 6.14), with an average household income of 25,900.44 baht per month (SD = 22,690.54). Most participants reported living with their partners (90.36%) and having easy access to low-fat foods (86.72%). Nearly three-fourths of those were nulliparous and had sufficient income. However, over half of them had overweight pre-pregnancy BMI, no medical conditions, early ANC visits, less than 8 ANC visits, and had not received GWG advice. Regarding health cognitions, over half of the participants had adequate knowledge, internal WLOC, high social support, and intention to control GWG; they had a low perceived barrier to gestational weight control. Likewise, 50.80% and 56.79% of pregnant women with overweight/obesity had appropriate healthy food consumption and adequate PA during pregnancy.

### Nurse-midwife's characteristics and information

Fifty-one NMs from 10 ANC units (from a response rate of 10 Head nurses and 46 NMs, 91.07%) completed the questionnaires. Participants' mean age and work experiences were 48.39 (SD = 8.755) and 9.31 years (SD = 8.76) respectively. Most participants earned bachelor's degrees and had more than ten years of work experience. Over three-fourths of NMs reported that they never received training on weight management for pregnant women with overweight/obesity. In addition, over half of the participants revealed they did not have sufficient GWG knowledge. The mean GWG knowledge, attitudes toward GWG control, and GWG counseling practice scores were 43.82 (SD = 5.344), 13.78 (SD = 1.316), and 51.20 (SD = 1.32), respectively. However, only 57.14% of participants in NUHs had positive attitudes toward GWG control. GWG counseling practice was non-routine in all UHs and in 5 NUHs (Table 2).

**Table 2:** Characteristics of nurse-midwives in 10 tertiary hospitals, Bangkok and Metropolitan area, Thailand, 2019

	**Type of hospital**		**Total, *n* = 51**
	**UHs, *n* = 16**	**NUHs, *n* = 35**	
**GWG knowledge** [n(%)]			
Sufficient	6(37.50)	17(48.57)	23(45.10)
Insufficient	10(62.50)	18(51.43)	28(54.90)
**Attitudes toward GW control** [n (%)]			
Positive	16(100)	20(57.14)	36(70.59)
Negative	0(0)	15(42.86)	15(29.41)
**GWG counseling practice** [n (%)]			
Routine	0(0)	30(85.71)	30(58.82)
Non-routine	16(100)	5(14.29)	21(41.18)

*Abbreviations*: *n* number

### ANC service arrangement for pregnant women with overweight/obesity

The participating hospitals included eight NUHs and two UHs. There were 114 cases (SD = 40.26) per day. The healthcare providers were obstetricians (OBs), NMs, nurse assistants, and advanced practice nurses (APNs). ANC service arrangements in all UHs provided ANC service every weekday and had Maternal Fetal Monitoring (MFM) clinics available. The weight management service for pregnant women with overweight/obesity are not available in all tertiary care. In two UHs and two NUHs, the ANC providers (OBs and NMs) provided GWG counseling for pregnant women with overweight/obesity when they performed abdominal examinations. If the gestational weight was uncontrolled, the ANC providers cooperated with the dietitians or APNs for GWG counseling practice. In the fewer NUHs, GWG counseling was provided by NMs or general physicians, and group health education on nutrition and diet control by the dieticians. In addition, seven out of eight NUHs had nurse-patient ratios more than standard criteria (1:20). Moreover, various pre-pregnancy BMI and GWG guidelines were used in each hospital. The dietary and PA guidelines were not available in most tertiary care. However, the quality of general ANC service provision in tertiary care remained at a good to excellent level. Some NUHs did not use a nutrition graph or weight gain chart plotting to evaluate nutrition and weight gain status. Specific ANC services were provided at a good level in two UHs and four NUHs (Table 3).

**Table 3:** ANC service delivered in 10 tertiary hospitals, Bangkok and Metropolitan area, Thailand, 2019

	**Type of hospital**		**Total, *n* = 10**
	**UHs, *n* = 2**	**NUHs**, ***n* = 8**	
**Using pre-pregnancy BMI and GWG guidelines** [n (%)]			
No	2(100)	8(100)	10(100)
**Using dietary and PA guidelines** [n (%)]			
Yes	2(100)	1(12.5)	3(30)
No	0(0)	7(87.5)	7(70)
**Quality of general ANC service provision** [n (%)]			
Very good	2(100)	2(25)	4(40)
Good	0(0)	5(62.5)	5(50)
Fair	0(0)	1(12.5)	1(10)
**Quality of specific ANC service provision** [n (%)]			
Good	2(100)	4(50)	6(60)
Fair	0(0)	4(50)	4(40)

### Predicting factors and impacts of inappropriate GWG

The pregnant women with overweight/obesity who had sufficient income (AOR 0.49; 95% CI: 0.27–0.92) and ease of access to low-fat food (AOR 0.31; 95% CI: 0.13–0.77) had significantly decreased odds of inadequate GWG than those without such resources and access. Paradoxically, the women who had adequate GWG knowledge were 1.81 times (AOR 1.81; 95% CI: 1.02–3.23) more likely to have inadequate GW than women with inadequate GWG knowledge. GWG counseling practice by ANC providers as a part of general ANC services (AOR 0.03; 95% CI: 0.00–0.53) were assessed to be of good (AOR 0.02; 95% CI: 0.00–0.63) or very good quality (AOR 0.01; 95% CI: 0.00–0.39), with positive attitudes toward GWG control by healthcare providers (AOR 0.20; 95% CI: 0.06–0.61). These practices and attitudes significantly decreased the odds of inadequate GWG. ANC service system factors were not significantly associated with excessive GWG. Meanwhile, only maternal predicting factors, namely the convenience of access to low-fat food (AOR 0.29; 95% CI: 0.14–0.60) and internal WLOC (AOR 0.57, 95% CI 0.37–0.86), significantly decreased the odds of excessive GWG (Table 4).

**Table 4:** Predicting factors of GWG of tertiary hospitals in Bangkok and Metropolitan area, Thailand, 2019

	**Inadequate GWG, *n* = 89**				**Excessive GWG, *n* = 427**			
	**COR**	**95%CI**	**AOR**	**95%CI**	**COR**	**95%CI**	**AOR**	**95%CI**
**Maternal Factors**								
Overweight	0.85	0.49–1.47	0.87	0.48–1.56	**0.67**	**0.46–0.99**^a^	0.74	0.49–1.12
Sufficiency income	**0.44**	**0.25–0.79**^a^	**0.49**	**0.27–0.92**^a^	0.74	0.47–1.17	0.80	0.50–1.29
Living with partner	0.86	0.40–1.85	0.80	0.40 -1.98	1.55	0.87–2.78	1.73	0.93- 3.20
Multiparous	1.03	0.56–1.86	1.02	0.55–1.91	1.35	0.90–2.03	1.44	0.93–2.21
Easy access to low-fat food	**0.28**	**0.12–0.66**^a^	**0.31**	**0.13–0.77**^a^	**0.30**	**0.15–0.62**^a^	**0.29**	**0.14–0.60**^a^
Received GWG advice	0.59	0.35–1.01	0.63	0.35–1.13	1.04	0.72–1.48	1.33	0.90–1.98
Adequate GWG Knowledge	1.71	0.99–2.97	**1.81**	**1.02–3.23**^a^	0.88	0.61–1.27	0.89	0.60–1.30
A low perceived barrier to GWG control								
	1.26	0.75–2.10	1.20	0.69–2.08	**1.50**	**1.05–2.15**^a^	1.44	0.98–2.11
Internal WLOC	0.77	0.44–1.35	0.80	0.44–1.43	**0.62**	**0.42–0.91**^a^	**0.57**	**0.37–0.86**^a^
Appropriate Healthy food consumption								
	0.83	0.50–1.39	0.89	0.51–1.56	0.77	0.54–1.10	0.90	0.61–1.33
**The ANC service system factors**								
Using Dietary and PA guidelines								
	0.77	0.46–1.31	5.45	0.92–32.13	**0.67**	**0.47–0.97**^a^	0.97	0.27–3.47
Nurse-patient ratio < 1: 20	0.64	0.36–1.17	3.05	0.90–10.32	0.81	0.52–1.26	1.33	0.54–3.32
GWG counseling by ANC providers								
	1.29	0.51–3.29	**0.03**	**0.00–0.53**^a^	1.89	0.99–3.64	0.36	0.04–3.66
Excellent general ANC service quality								
	0.83	0.15–4.73	**0.01**	**0.00–0.39**^a^	**0.23**	**0.08–0.66**^a^	0.07	0.00–1.41
Good general ANC service quality								
	1.36	0.24–7.74	**0.02**	**0.00–0.63**^a^	**0.29**	**0.10–0.85**^a^	0.10	0.01–1.30
Good specific ANC service quality								
	0.63	0.38–1.06	0.28	0.08–1.04	0.88	0.61–1.25	0.65	0.25–1.69
Positive attitude to control the weight by the provider								
	0.44	0.24–0.83	**0.20**	**0.06–0.61**^a^	0.78	0.48–1.27	0.55	0.23–1.33
Routine GWG counseling practice								
	1.42	0.84–2.41	1.38	0.14–13.59	1.41	0.99–2.03	0.85	0.16–4.35

*Reference category*: adequate gestational weight gain, ^a^statistically significant at level 0.05

adjusted for type of hospital, using pre-pregnancy BMI and GWG guidelines, ANC provider's knowledge, maternal age, medical condition, social support, intention to control GWG, and physical activity

Pregnant women with overweight/obesity who had excessive GWG were more likely to have primary C/S (AOR 1.65; 95% CI: 1.07–2.55), fetal LGA (AOR 1.60; 95% CI: 1.01–2.54), and macrosomia (AOR 5.84; 95% CI: 1.38–24.80), compared to those with adequate GWG. Meanwhile, those pregnant women who had inadequate GWG were not significantly associated with SGA (AOR 1.07; 95% CI: 0.55–2.07) (Table 5).

**Table 5:** Impacts of inappropriate GWG in tertiary hospitals in Bangkok and Metropolitan area, Thailand, 2019

	**Type of GWG overall**			**IGWG vs AGWG, *n* = 258**		**EGWG vs AGWG, *n* = 596**	
	**AGWG, *n* = 169**	**IGWG, *n* = 89**	**EGWG, *n* = 427**	**OR (95%CI)**	***p*-value**	**OR (95%CI)**	***p*-value**
**Primary C/S** ^a^							
Yes	33 (19.50)	21(23.60)	122(28.60)	NA	NA	**1.65(1.07–2.55)**	0.024*
No	136 (80.50)	68 (76.40)	305(71.40)				
**PIH** ^b^							
Yes	7(19.40)	5(13.90)	24(66.70)	NA	NA	1.28(0.5–3.26)	0.465
No	162(25.00)	84(12.90)	403(62.10)				
**SGA**							
Yes	137(27.70)	73(14.70)	285(57.60)	1.07 (0.55–2.07)	0.851	NA	NA
No	32(16.90)	16(8.50)	141(74.60)				
**LGA**							
Yes	28(19.90)	10(7.10)	103(73.00)	0.64(0.29–1.38)	0.253	**1.60(1.01–2.54)**	0.046*
No	141(25.90)	79(14.50)	324(59.60)				
**Macrosomia** ^c^							
Yes	2(5.90)	2(5.90)	30(88.20)	NA	NA	**5.84(1.38–24.80)**	0.017*
No	167(25.70)	87(13.40)	397(61.00)				

^a^adjusted for NMs’ knowledge, NMs’ attitude, GWG counseling by ANC providers, parity, and maternal’ GWG knowledge

^b^adjusted for nurse-patient ratio, social support, and healthy food consumption

^c^adjusted for pre-pregnancy BMI

*Reference category*: Adequate GWG, * statistically significant at 0.05

## Discussion

The study's findings indicated that the prevalence of inappropriate GWG among pregnant women with overweight/obesity in Bangkok and metropolitan areas, Thailand is still high (75.33%), especially for excessive GWG. This finding is congruent with previous studies that have reported women who are overweight and obese are more likely to gain excess weight as recommended by the IOM guidelines [2, 6, 10]. A high rate of excessive GWG might be because the recommendation of total GWG by IOM for pregnant women with overweight/obesity (2009) [8] is narrow. Thus, it is difficult for them to control. Moreover, appropriate weight gain had multiple influences such as personal and environmental factors, and advice from healthcare providers [44]. However, GWG tends to decline as pre-pregnancy BMI increases [45].

This study illustrates that ANC service system arrangements for preventing inappropriate GWG for pregnant women with overweight/obesity are similar to those for normal-weight pregnant women. There was no specific intervention for weight management in all hospitals. Pregnant women with overweight/obesity received care in high-risk clinics only when they had complications or maternal morbidity to prevent adverse maternal and neonatal outcomes. In addition, there were own guidelines on weight management, and some guidelines were not regularly used in the care of pregnant women with overweight/obesity. Even though most NMs had self-assessed adequate knowledge and positive attitudes toward controlling GW, they had never received training in GWG management. Consequently, GWG counseling was only provided as a routine practice. Therefore, the GWG counseling may not have been personalized to women's needs or specific to pregnant women with overweight/obesity. Moreover, the GWG information provided was only general nutrition education due to a high workload or less nurse-patient ratio in each ANC unit. Thus, healthcare providers could provide the GWG information only as a group nutrition education.

These findings are congruent with previous studies that showed that each ANC unit in Bangkok, Thailand had different approaches to providing services and developed clinical practice guidelines regarding three main services (antenatal screening, disease treatment, and health education) based on recommendations of the Department of Health and the Royal Thai College of Obstetricians and Gynecologists [46]. Sindhu et al. (2017) pointed out that pregnant women having complications should receive care in high-risk clinics at tertiary care [37]. Although, there is no evidence to support that specific clinics for pregnant women with overweight/obesity are needed. Several studies found that the service delivery for controlling gestational weight among pregnant women with overweight/obesity should be designed as adaptive interventions/dosages throughout periods of pregnancy [28, 29]. Previous study pointed out that GWG control should be individual face to face counseling 45–60 min by professional ANC providers, starting with education on GWG/PA/energy intake (EI), and importance of GWG related factors. The tailored goal-setting/action plans for achieving GWG/PA/EI goals can be performed, as well as self-monitoring of GWG with mHealth devices, assessing and monitoring fetal growth and development [30]. Moreover, adequate and targeted information about optimizing nutrition, lifestyle, and weight gain in pregnancy is significantly associated with women’s compliance with IOM guidelines [22, 28].

Multivariate logistic analysis showed that maternal factors and the antenatal care service system could jointly predict a likelihood of inappropriate GWG with a Nagelkerke' s Pseudo R-Square of 0.1406. Adequate GWG knowledge, sufficient income, easy access to low-fat food were the significant predictors of inadequate GWG at the individual level. This finding aligns with the previous reports that appropriate GWG was associated with maternal and environmental factors [8, 47, 48]. An inadequate weight gain is protective against LGA for class 2 and 3 obese pregnant women and GDM for the class 3 obesity group [49]. Adequate GWG knowledge among pregnant women with overweight/obesity affected inadequate GWG. This might be because they were concerned and attempted to control their body weight to prevent adverse health outcomes such as GDM and fetal LGA. Sufficient income and easy access to low-fat food enabled women to purchase nutritious food and practice a healthy diet for weight control. Previous evidence also showed that access to healthy food [8, 50] and sufficient income [11] are facilitators of adequate GWG. However, easy access to healthy food as low-fat food was also significantly associated with excessive GWG. The high-fat food restriction is an essential topic of GWG counseling [51], while the gestational fatty acid status is essential for healthy fetal development [52]. According to lifestyle interventions based on nutritional concepts, most obstetricians and nurse-midwives in tertiary hospitals recommended low-fat food to control gestational weight to prevent adverse maternal and neonatal outcomes. Likewise, a previous study revealed that an effective lifestyle intervention (increases the proportion of polyunsaturated fatty acids and omega-6 fatty acids from a low-fat food diet) is unlikely to have an adverse impact on the developing fetus [53]. Moreover, the internal WLOC was a vital health cognitive factor reflecting individuals' beliefs in their abilities to control body weight [16, 17]. Thus, only maternal factors such as WLOC and access to low fat food were predictive of excessive weight gain among pregnant women with overweight/ obesity. Moreover, the chi-square analysis on the factors associated with excessive gain were not differences in proportions in subjects from different locations in this study.

According to ANC service system factors in this study, the group of significantly protective factors with inadequate GWG were good to excellent general ANC service quality, the GWG counseling by ANC providers, and a positive attitude toward weight control of healthcare providers. This finding is consistent with previous reports that health interventions, provider advice, and health facilities were the factors in the ANC service delivery system related to GWG [8, 45]. Current general ANC service provisions cover standard antenatal care, including gestational weight gain monitoring and control activities were associated with good pregnancy outcomes [26, 36, 37]. GWG counseling practice delivered by a team of experienced ANC providers resulted in appropriate GWG as compared to interventions delivered by non-prenatal care providers [54]. With a team approach, women may receive clearer directives about the impact of inappropriate GWG and how to control their weight. Most NMs in this study had positive attitudes toward gestational weight control. As in a previous study, nurse-midwives should encourage pregnant women with overweight/obesity to control GWG [21].

Birth outcomes such as risk of primary cesarean section, fetal LGA, and macrosomia were significantly associated with GWG. These findings are congruent with previous studies indicating that pregnant women with overweight/obesity who gained gestational weight excessively were more likely to have a cesarean delivery because of delayed cervical dilation, shoulder dystocia, and macrosomia [1, 2, 6].

The current ANC service arrangement can prevent inadequate weight gain but not excessive weight gain because there was overcrowding in the ANC unit, limited knowledge, and insufficiently trained healthcare providers. Quality of ANC service provision at good to very good level and a positive attitude among healthcare providers to control gestational weight were key predicting factors preventing inappropriate GWG among the study population. Therefore, a training program for GW management should be established to ensure self-confidence among ANC providers in GWG counseling practice. Moreover, an evaluation and monitoring process for the quality of ANC services should be undertaken to improve maternal and neonatal outcomes.

### Strengths and limitations

Our findings identify previously unconfirmed predictive factors regarding the prevalence of inappropriate GWG among pregnant women with overweight/obesity and ANC service system arrangements in Bangkok, Thailand. This research extends knowledge regarding GWG management for pregnant women with overweight/obesity living in the urban areas of Thailand. These findings as preliminary evidence call for establishing gestational weight policy and guidelines for vulnerable pregnant women. However, a limitation of this study is the possible bias of self-reported data of some factors due to the research instruments. The researcher selected the best research instruments based on national and international guidelines and a literature review from a standardized database system to modify and develop the most reliable research instruments.

## Conclusion

In conclusion, inappropriate GWG among pregnant women with overweight/obesity in metropolitan Bangkok remained high, especially for excessive GWG that is significantly associated with primary C/S, fetal LGA, and macrosomia in the health care system. ANC service provisions covered at least the standard antenatal care recommendations and guidelines. In addition, GWG counseling was provided by ANC providers during physical examination and affected women's intention to control gestational weight. Moreover, the positive attitude of nurse-midwifes who provided good to excellent quality ANC service influenced appropriate GWG among pregnant women with overweight/obesity. Thus, good ANC service arrangements can reduce the prevalence of inappropriate GWG for pregnant women with overweight/obesity. However, a training program for GW management should be established to ensure knowledge self-confidence among ANC providers in GWG counseling practice.

## Data Availability

The datasets used and/or analyzed during the current study available from the corresponding author on reasonable request.

## Abbreviations

GWG: Gestational weight gain
ANC: Antenatal care
NMs: Nurse-midwives
BMI: Body mass index
WLOC: Weight locus of control
MOPH: Ministry of Public Health in Thailand
UHs: University hospitals
NUHs: Non-University hospitals
